# Supplementary material for: Transcriptomic Insights into Mechanisms of Early Seed Maturation in the Garden Pea (Pisum sativum L.)
Source: Cells. 2020 Mar 23;9(3):779. doi: 10.3390/cells9030779 (PMC7140803; doi:10.3390/cells9030779)

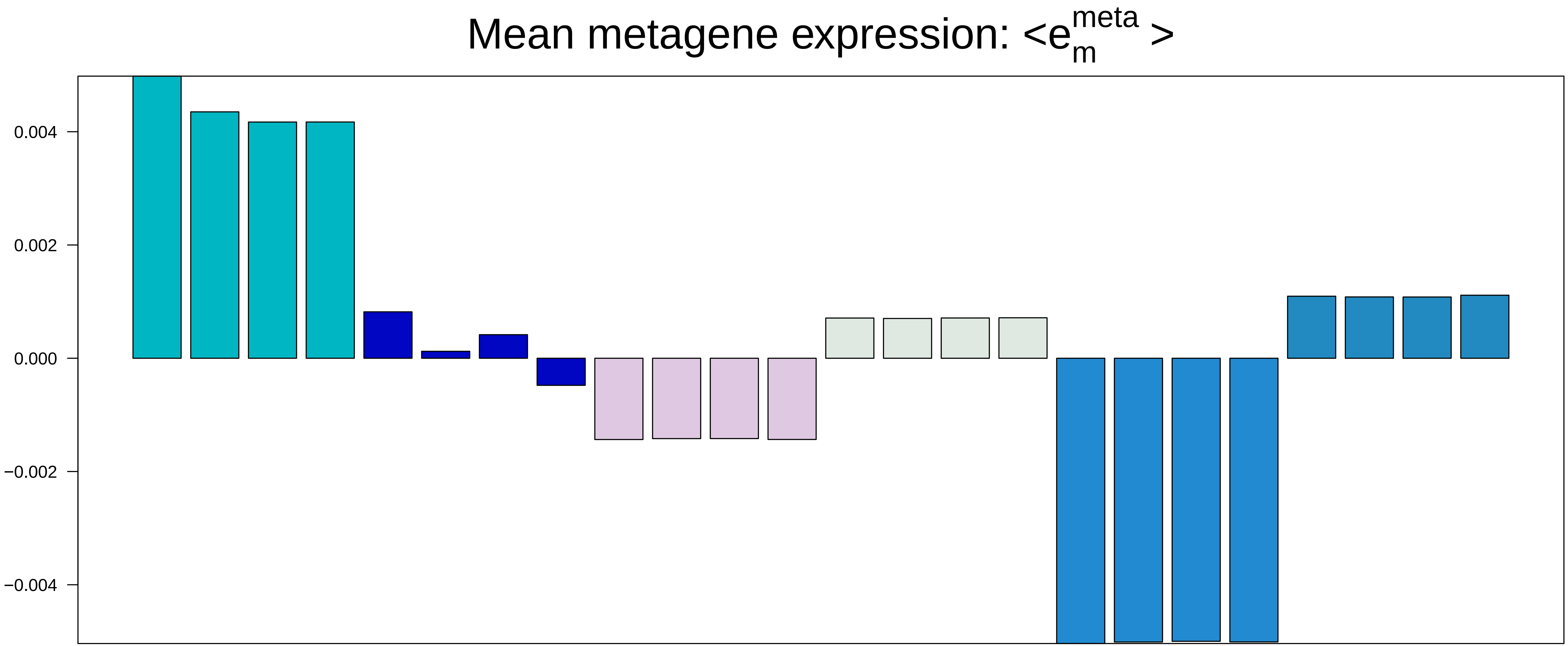
Mean metagene expression: $<e_m^{meta}>$
0.004
0.002
0.000
−0.002
−0.004

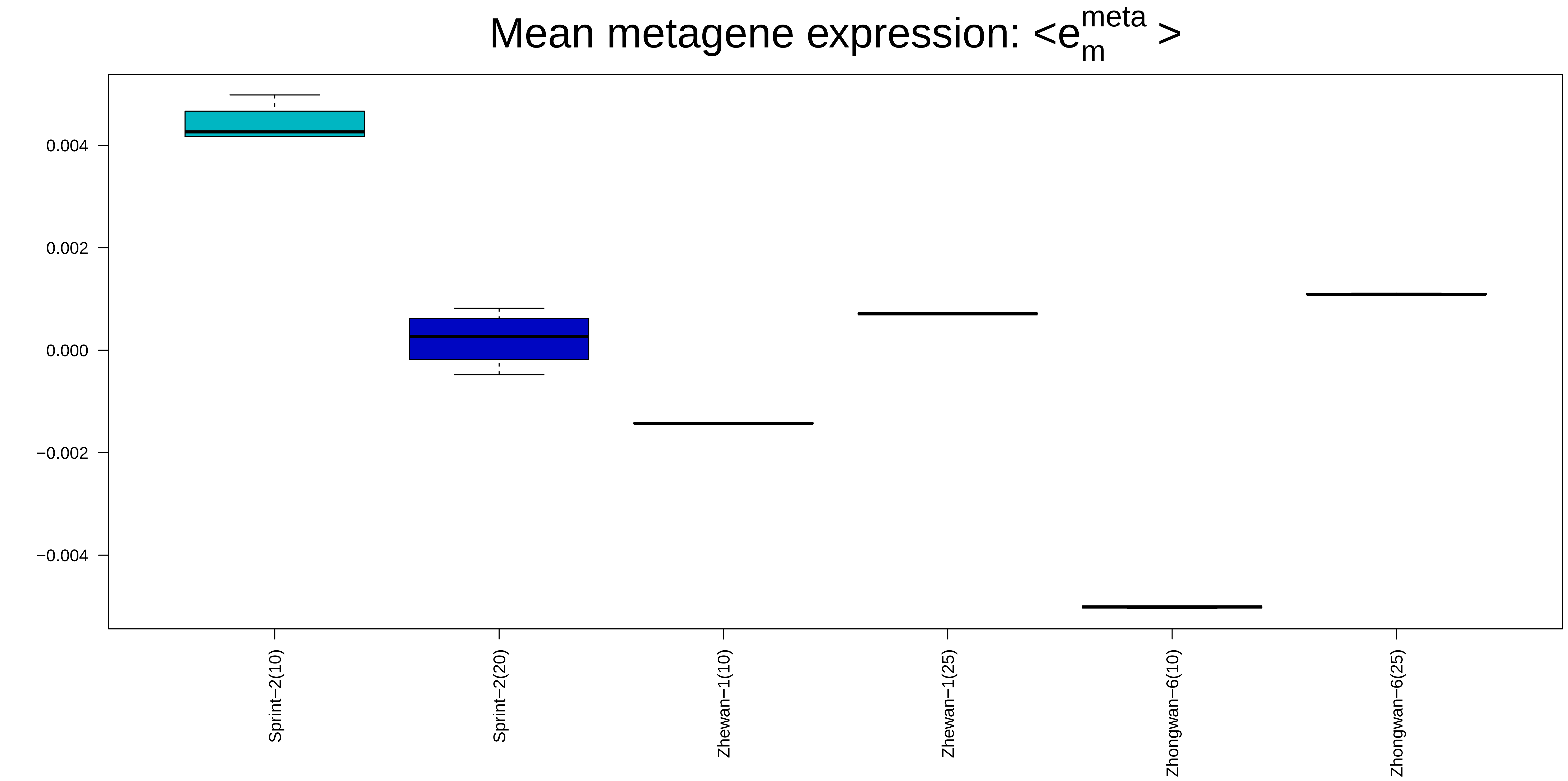

Mean metagene expression: $<e_m^{meta}>$
0.004
0.002
0.000
−0.002
−0.004
Sprint−2(10)
Sprint−2(20)
Zhewan−1(10)
Zhewan−1(25)
Zhongwan−6(10)
Zhongwan−6(25)

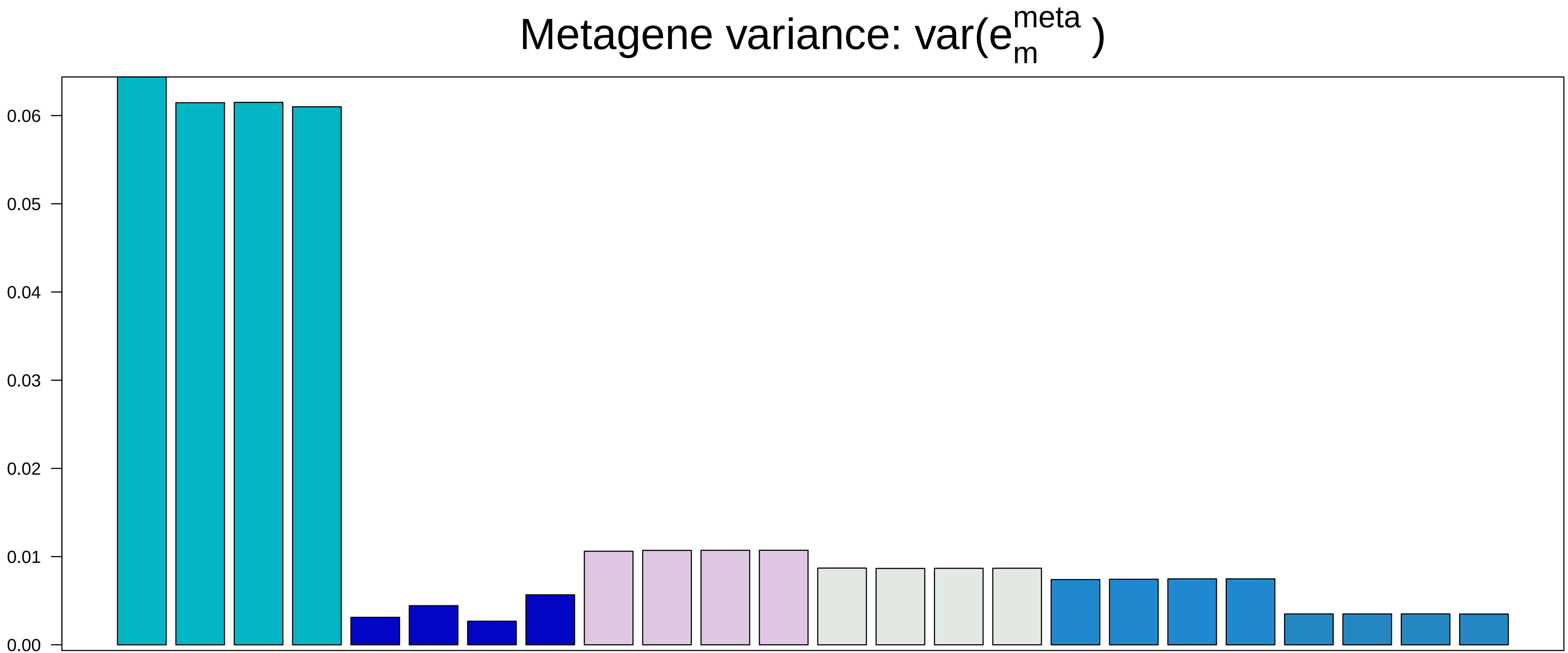
Metagene variance: var(e_m^{meta})
0.06
0.05
0.04
0.03
0.02
0.01
0.00

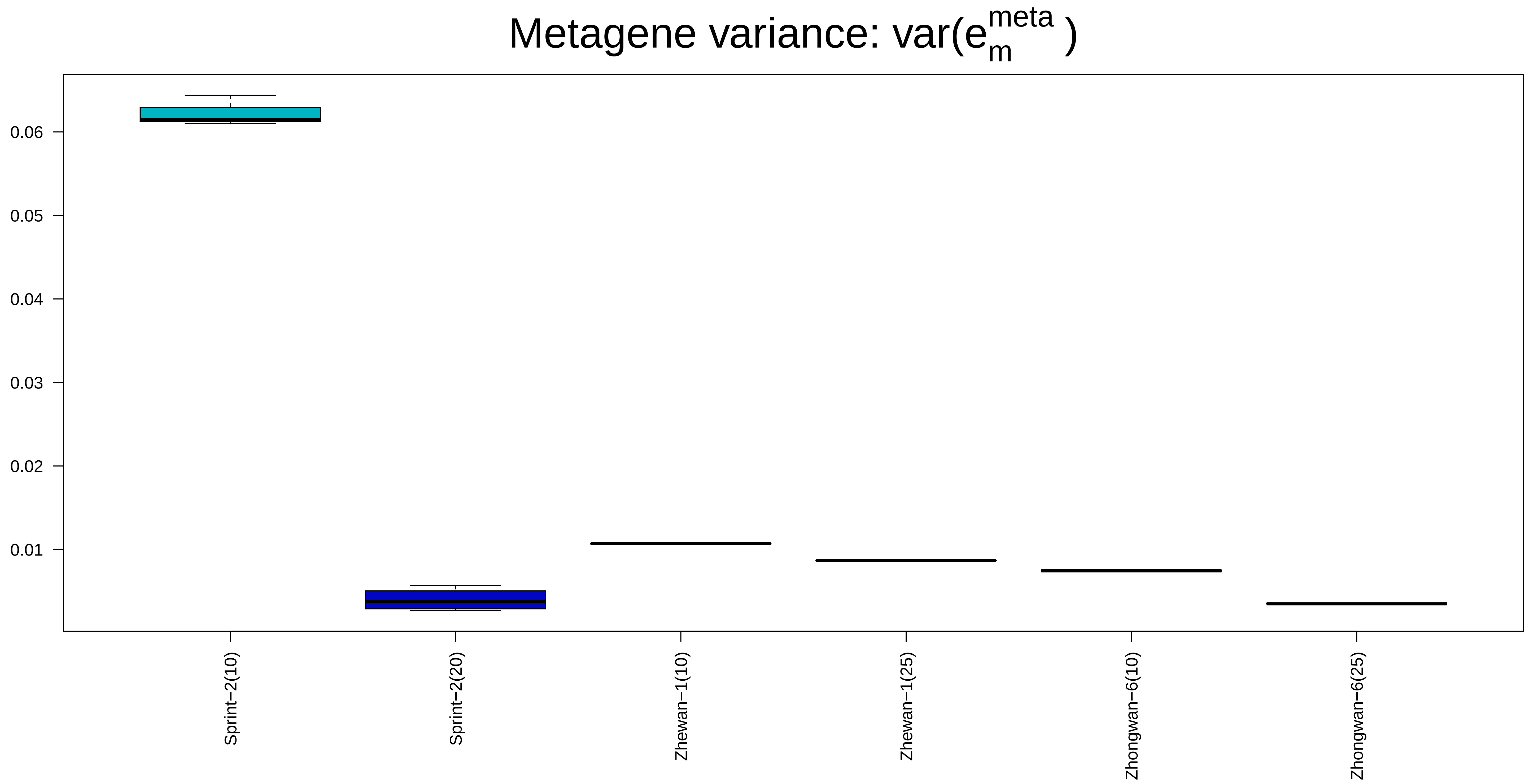
Metagene variance: var(e_m^{meta})
0.06
0.05
0.04
0.03
0.02
0.01
Sprint−2(10)
Sprint−2(20)
Zhewan−1(10)
Zhewan−1(25)
Zhongwan−6(10)
Zhongwan−6(25)

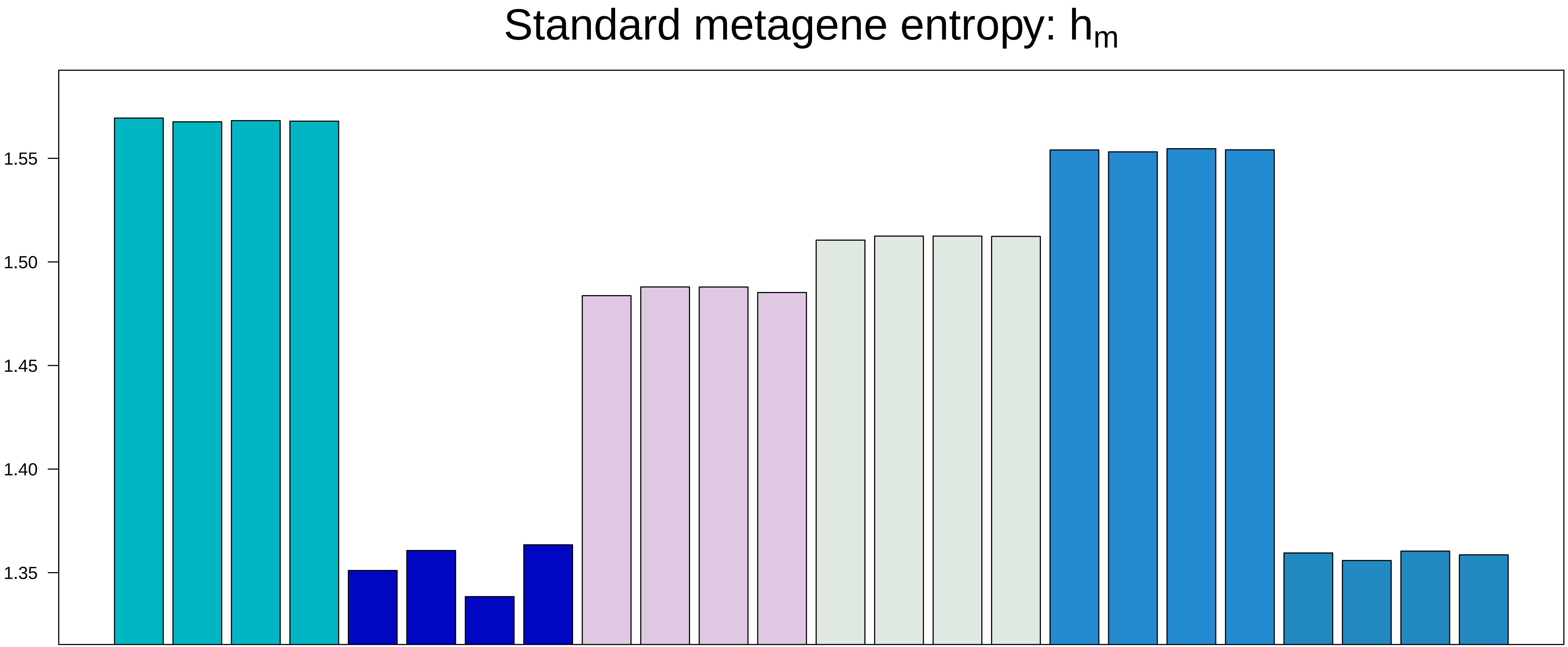
Standard metagene entropy: h_m
1.55
1.50
1.45
1.40
1.35

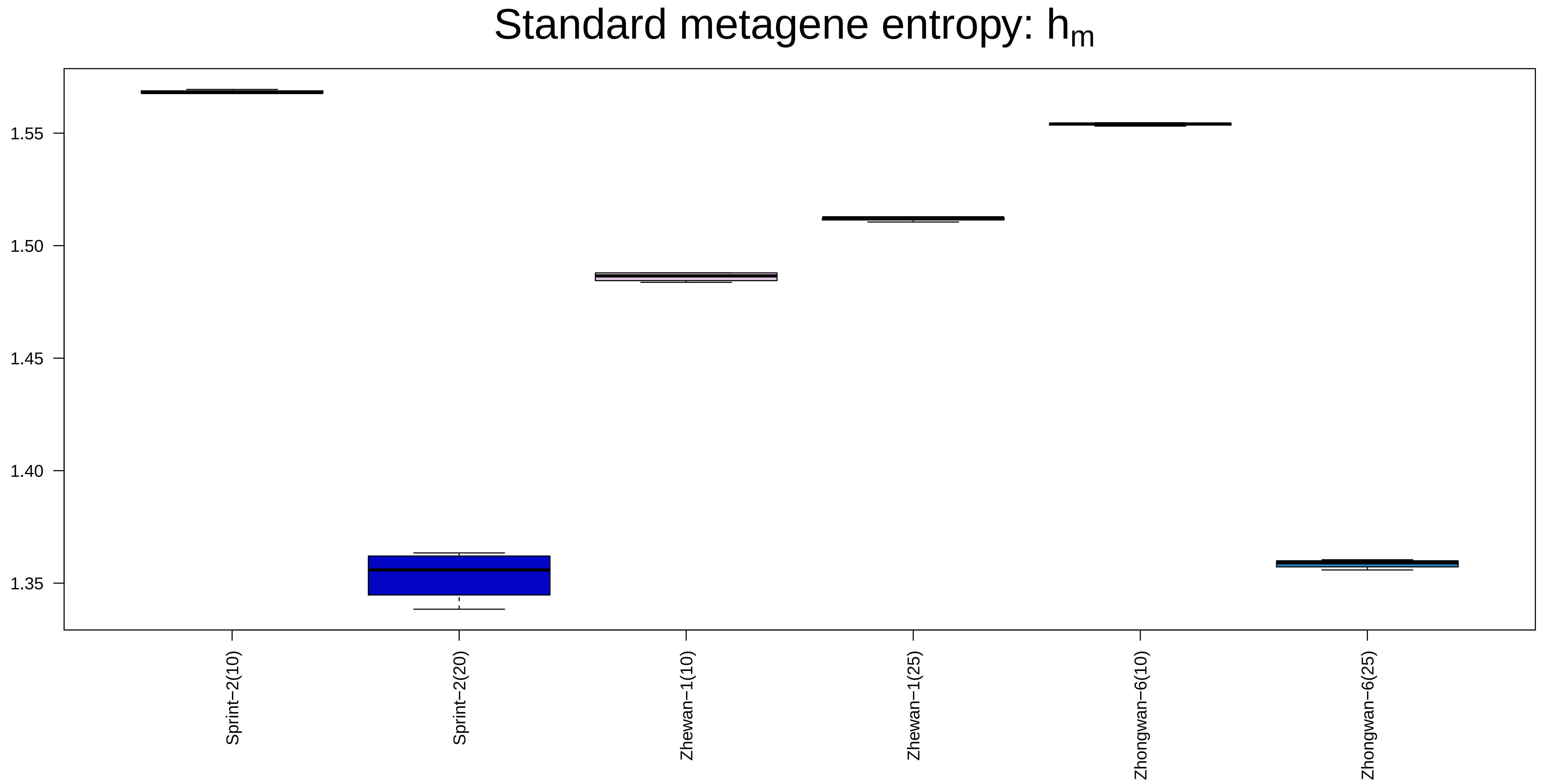

Standard metagene entropy: h_m
1.55
1.50
1.45
1.40
1.35
Sprint−2(10)
Sprint−2(20)
Zhewan−1(10)
Zhewan−1(25)
Zhongwan−6(10)
Zhongwan−6(25)

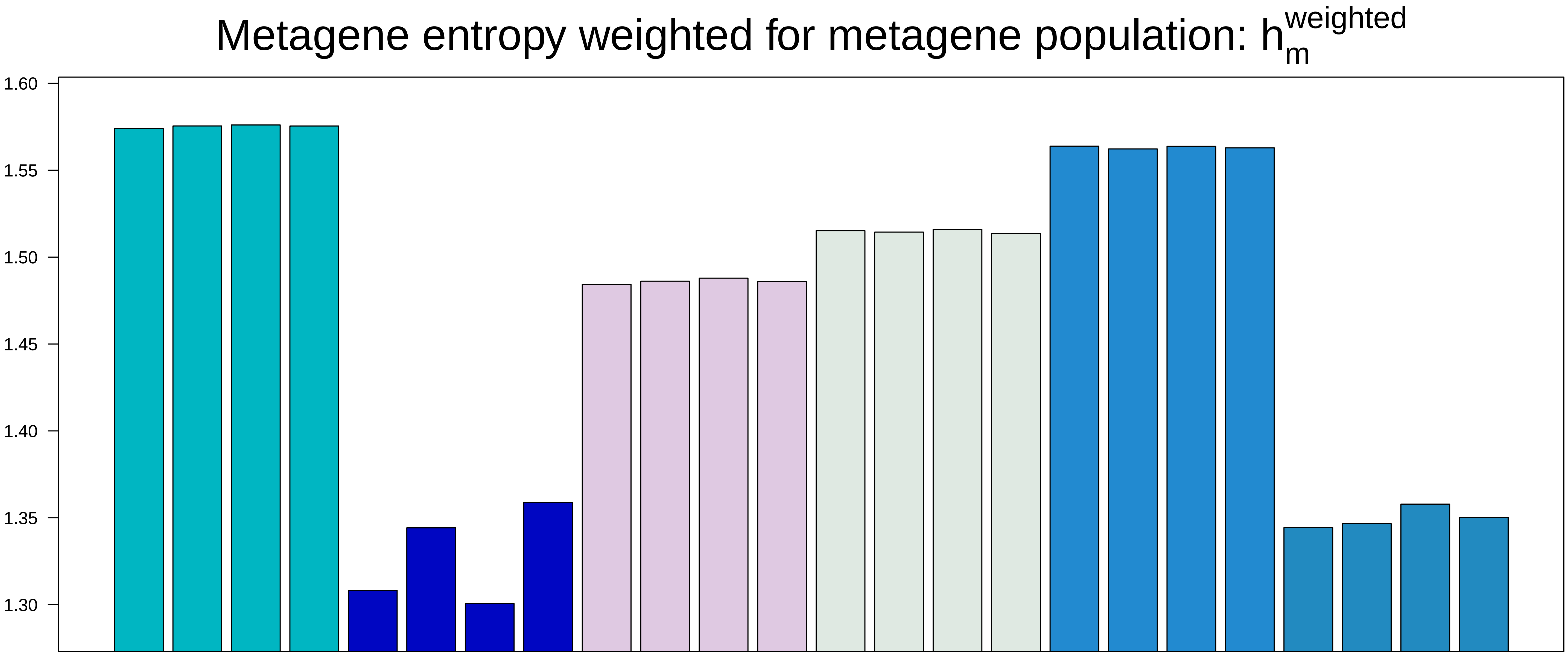

Metagene entropy weighted for metagene population: $h_m^{weighted}$
1.60
1.55
1.50
1.45
1.40
1.35
1.30

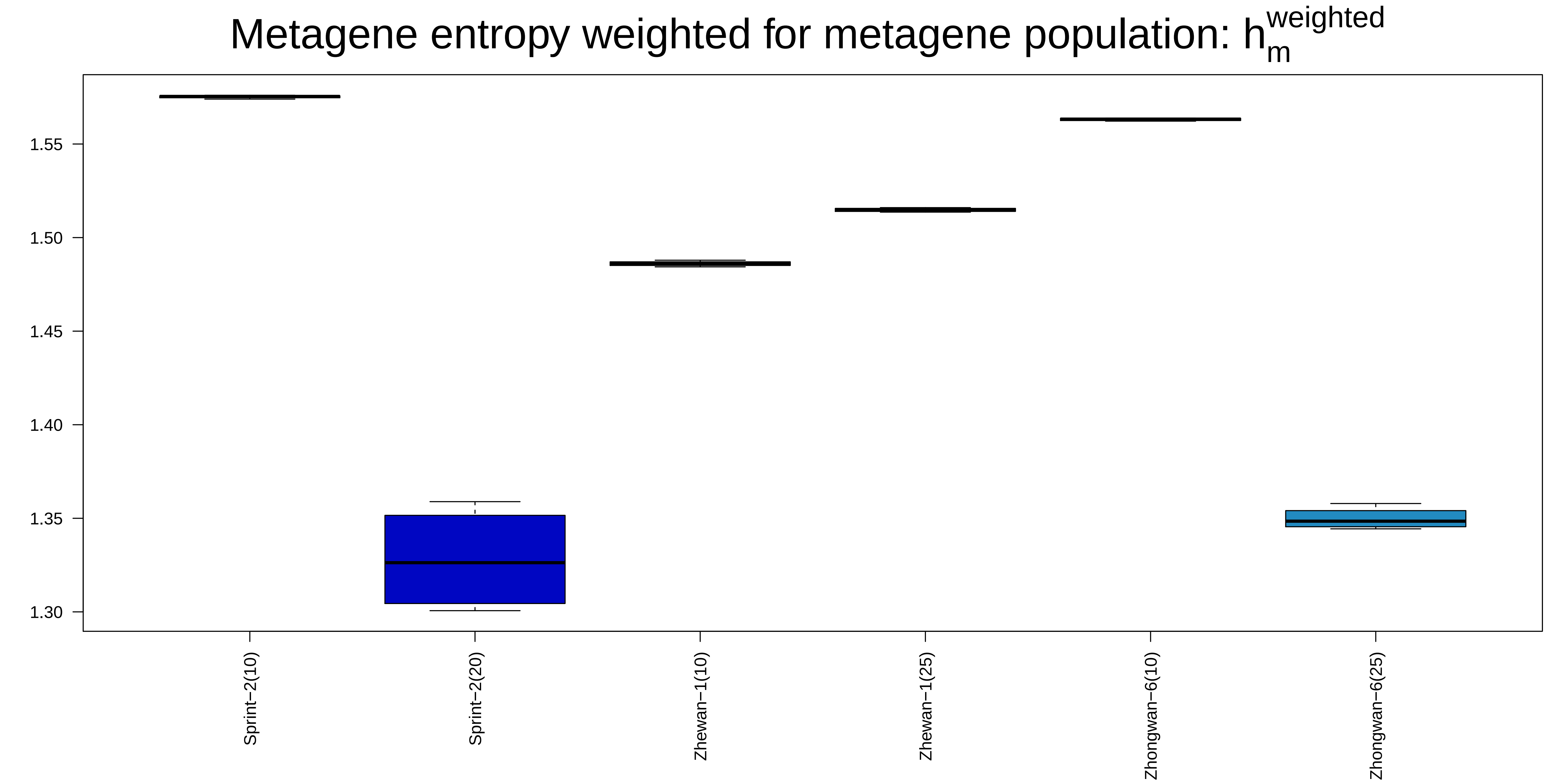
Metagene entropy weighted for metagene population: $h_m^{weighted}$
1.55
1.50
1.45
1.40
1.35
1.30
Sprint−2(10)
Sprint−2(20)
Zhewan−1(10)
Zhewan−1(25)
Zhongwan−6(10)
Zhongwan−6(25)

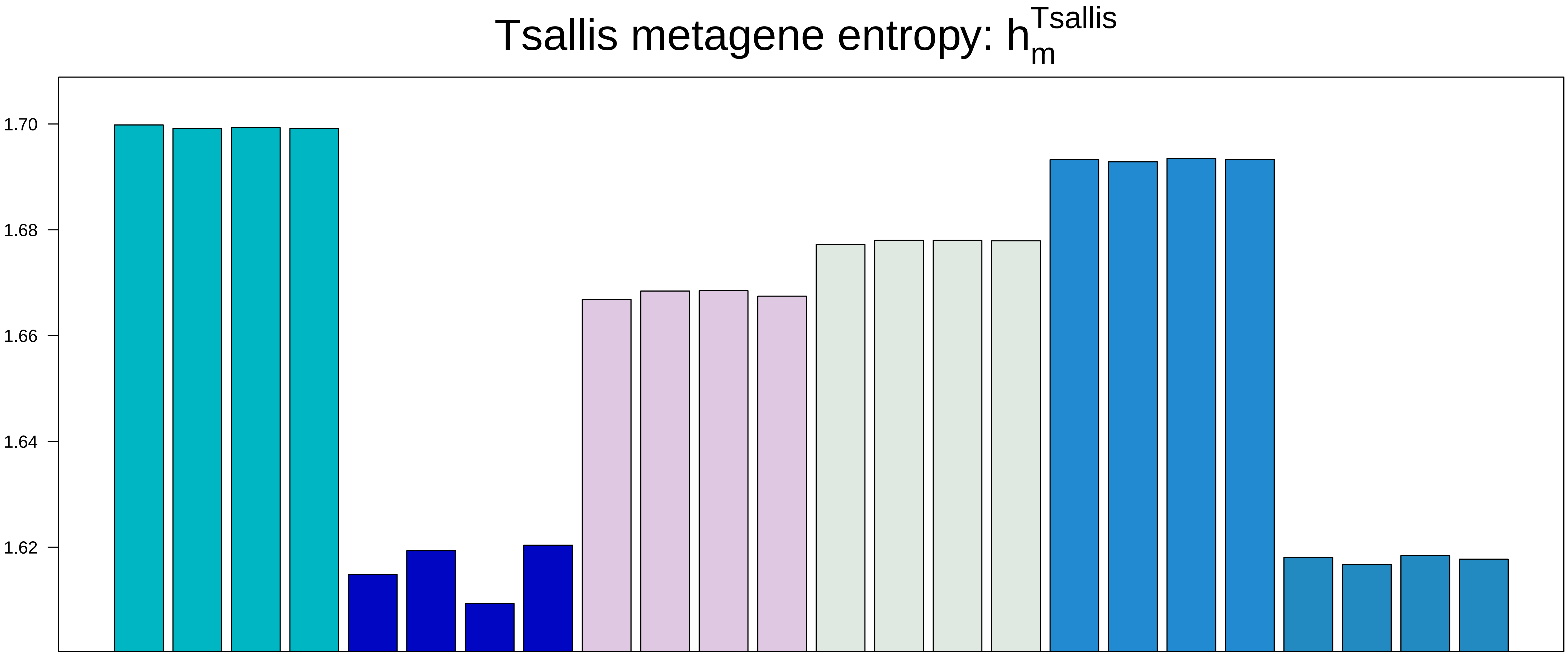
Tsallis metagene entropy: $h_m^{Tsallis}$
1.70
1.68
1.66
1.64
1.62

Tsallis metagene entropy: $h_m^{Tsallis}$

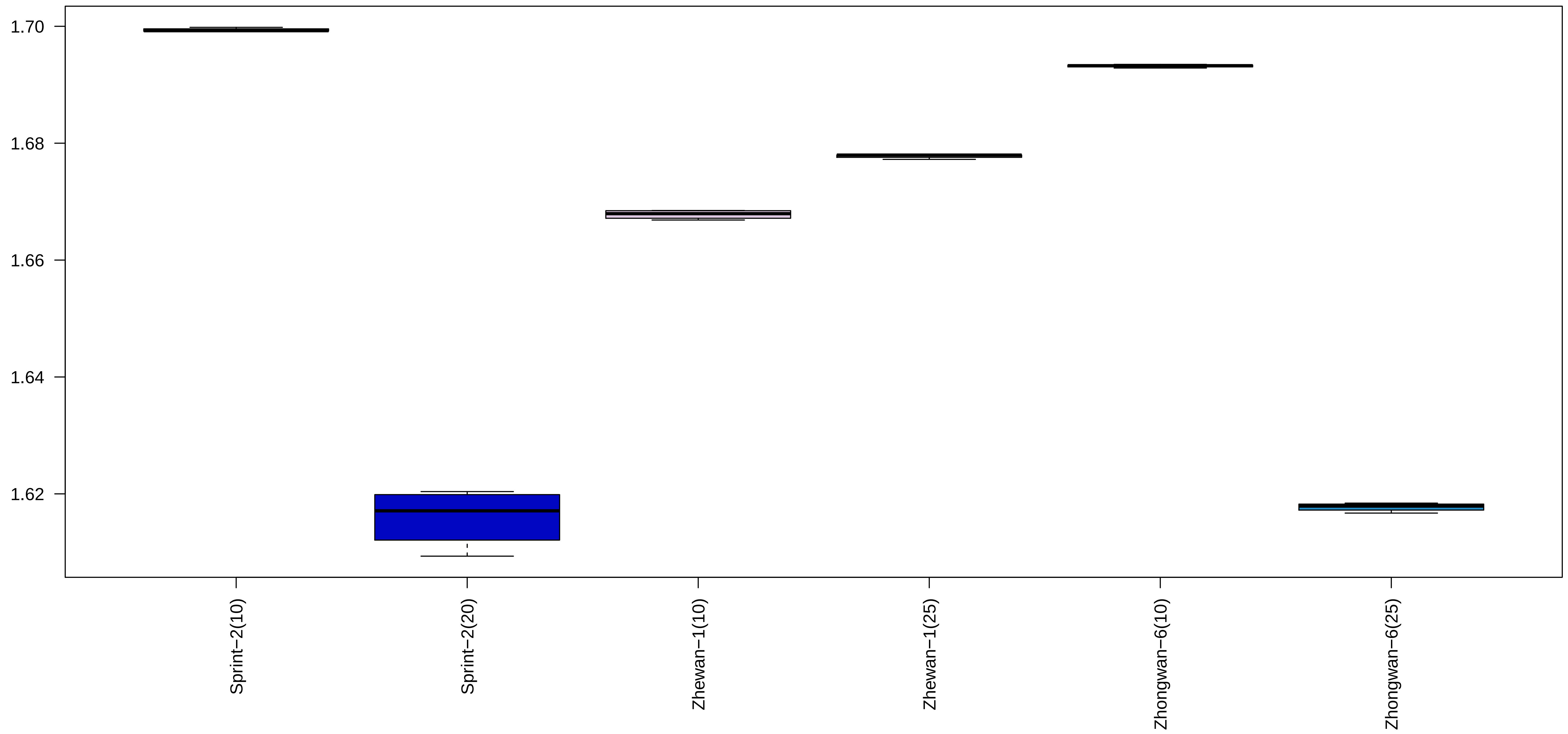

Supplement: Supplementary file 1 [file cells-09-00779-s001.zip › Supplementary materials/FileS2.zip!/FileS1/Supporting Maps&Profiles/Entropy Profiles.pdf]
